# Factors That Determine the Likelihood of Giving Birth to the First Child within 10 Months after Marriage

**DOI:** 10.1155/2020/4675907

**Published:** 2020-03-20

**Authors:** Abdul-Karim Iddrisu, Francis Kwame Bukari, Kwaku Opoku-Ameyaw, Gabriel Oppong Afriyie, Kassim Tawiah

**Affiliations:** ^1^University of Energy and Natural Resources, School of Sciences, Department of Mathematics and Statistics, Ghana; ^2^Kwame Nkrumah University of Science and Technology, Department of Mathematics, Ghana

## Abstract

**Background:**

One of the major aims of marriage is to procreate or give birth to a child. Childbirth is so crucial in marriage that it often determines the happiness of the couple. Too much delay in childbirth after marriage or the likelihood that one cannot give birth after marriage can lead to divorce. However, causes of delay in childbirth are often difficult to detect by both the Gynaecologist and the couple involved. This makes proposing solutions to issues related to childbirth usually unsuccessful.

**Methods:**

It is against this background that we conducted this study to identify factors that determine childbirth within 10 months or after 10 months of marriage (birth length) among women in Ghana. This was achieved by using a logistic regression model for the dichotomous birth length variable, adjusting for risk factors/predictors of birth length. The data used for the study were obtained from the 2014 Ghana Demographic and Health Survey, consisting 6,525 complete cases with 18 predictor variables. Statistical analyses were carried out using STATA version 14.1.

**Results:**

The results show that respondents who have ever terminated pregnancy are more likely (OR = 0.178, 95%CI = 0.044, 0.312) to deliver after 10 months, wives whose husbands have higher education are less likely (OR = ‐0.162, 95%CI = ‐0.236, ‐0.088) to give birth after 10 months of marriage, wives who reported that beating is justified if she goes out without her husband's notice are more likely (OR = 0.466, 95%CI = 0.305, 0.628) to give birth after 10 months, wives who reported that beating is justified if she neglects the child are more likely (OR = ‐0.305, 95%CI = ‐0.461, ‐0.149) to give birth within 10 months, and wives who reported that beating is justified when she argues with her husband are less likely (OR = ‐0.301, 95%CI = ‐0.451, ‐0.152) to give birth after 10 months of marriage. Every unit increase in the age of the respondent at marriage increases the likelihood of giving birth after 10 months of marriage, and a unit increase in the age of the respondent at first sex decreases the likelihood of giving birth after 10 months in marriage.

**Conclusions:**

For conception within 1 month of marriage, wives and husbands should/are encouraged to have frequent sex, any negative social behaviour or policies must be discouraged, experts' advice on contraceptive use must be sought, and women are encouraged to desist from termination of pregnancy at any time of their life. Husbands should openly express their desire and love for their children since this increases the likelihood of wives' desire to give birth. This leads to frequent sex, which then reduces conception time, and hence childbirth within the shortest possible time.

## 1. Background

It is known that procreation or childbirth is one of the main aims of marriage, and childbirth is so crucial in marriage that it often determines the happiness of the couple. Research on improving birth rates has received much attention following the success of *In Vitro Fertilization* (IVF) in 1979 [1]. The IVF consists of series of procedures used to help with fertility or prevent genetic problems and assists with the conception of a child. In the mid-1980s, various authors conducted research to investigate the psychosocial effects of infertility [2] and IVF treatment [3, 4]. These authors reported that infertility affects emotional well-being, satisfaction with life, and self-esteem [1]. Failure of measures, such as Assisted Reproductive Technology (ART), to improve fertility is associated with reduced life satisfaction and self-confidence as well as substantial psychological distress [1].

One may hypothesize that pregnancy and parenthood experienced after infertility and assisted conception will be very appealing. However, it has been shown that past infertility and ART conception are likely to be associated with higher anxiety about pregnancy loss, delayed mother-infant attachment, reduced maternal confidence, hypervigilant and overprotective parenting, and idealized expectations of parenting capacity and the infant [1]. Clinical reports supported with qualitative studies indicated that pregnancy and parenting may be more complex psychologically after assisted than spontaneous conception [5, 6]. For methods to improve chances of conception and childbirth, others resort to rituals such as prayers, charms, and amulets [7].

The implication is that too much delay in childbirth after marriage or when it is likely that one cannot give birth after marriage can lead to divorce [8]. However, causes of delay in childbirth are often difficult to detect by both the Gynaecologist and the couple involved, making treatment unsuccessful. What makes the delay in childbirth after marriage even more problematic is the difficulty associated with efforts to detect/determine the cause of such delay. Most often, couples tend to blame each other. In most cases, even the Gynaecologist is required to use various methods to identify the cause of delay in childbirth with various approaches/solutions to address the issues identified to be related to the delay in childbirth. Some of the causes (such as social factors) of delay in childbirth cannot be detected by any Gynaecological examination. It may be much easier to detect and propose solutions to issues that can be detected through medical examination. However, for social factors/causes, it may be very difficult to detect because only the couple involved know that such factors exist in their marriage. The worse of this is that the couple may not report such factors to a Gynaecologist if they are not asked directly by the Gynaecologist. Most often, society rarely considers social factors as potential causes of delay in childbirth.

Hammarberg and colleagues [1] study reviewed the available evidence of the psychological and social consequences of pregnancy, childbirth, and early parenting after assisted conception systematically. Various authors [9–11] revealed that there is a positive association between women's empowerment and some aspects of their health, such as fertility and contraception. Prata and colleagues' [10] research provided evidence of the relationship between women's empowerment and pregnancy or childbirth, including abortion. A research conducted in Northern China revealed a significant association between women's infertility incidence with their BMI, state of exercise, amount of menstrual flow, number of pregnancies, and number of abortions and among men, both staying up late and engaging in high-temperature occupations are independent factors affecting their fertility [12]. Rakesh and colleagues [13] revealed that lifestyle factors, such as the age at which to start a family, nutrition, weight, exercise, psychological stress, environmental, and occupational exposures, are associated with fertility. Lifestyle factors such as cigarette smoking, illicit drug use, and alcohol and caffeine consumption can negatively influence fertility [13].

In Ghana, the increase in the level of contraceptive use is one of the main causes of reduced fertility [14]. Other factors affecting fertility indirectly include age of woman, education, religion, place of residence, and child mortality experience. There is no enough and affordable high-quality infertility services in Ghana [8]. As a result, most women seek to improve their fertility by resorting to methods such as traditional healing, witchcraft, and spiritual mediation [8]. Severe sociocultural and economic challenges increase the rate of infertility among women in Ghana, and hence, there is the need for accessible and affordable high-quality infertility care in Ghana [8].

In this paper, we explored the effects of sociocultural and socioeconomic factors that are likely to influence conception time among women after marriage in Ghana. Hence, we structured the paper into four main sections as follows. We have already given the background of the study in Section 1. We introduce the study setting, size and source of data, and statistical methodologies used in Section 2.  Section 3 presents the results of the statistical analyses using the data. We discussed the results and gave concluding remarks in Section 4.

## 2. Methods

In this section, we introduce the study setting and the source of data. We also introduce the outcome variable of interest in this study, as well as factors that determine the value or the status of this outcome variable. Finally, we discuss statistical approaches used in this study.

### 2.1. Study Setting and Data Source

This study is conducted in Ghana, and the data used for the study are obtained from the Ghana Demographic and Health Survey for 2014. This is a cross-sectional study, where the outcome variable of interest and its associated risk factors were measured at a single time point. In this study, we focused on individual birth record data, obtained from the respondents during the survey. We observed that some of the individual records have missing values [15–17], and hence, analyses were restricted to only the complete cases [17]. The complete data consist of 6,525 individuals who provided responses on how long it takes for them to give birth to their first child after marriage. Using these data, we categorised individuals into two groups (birth length status); that is, those who gave birth to their first child within 10 months after marriage and those who gave birth after 10 months of marriage. Also, we consider data on factors that are likely to predict the duration of birth after marriage (birth length status). Ethical approval and consent to participate statements can be found on http://dhsprogram.com/What-We-Do/Protecting-the-Privacy-of-DHS-Survey-Respondents.cfm, approved by the ICF International Institutional Review Board (IRB). We will now introduce the outcome variable and the risk factors of birth length status.

### 2.2. Outcome Variable

In this study, the outcome variable of interest is birth length status (which takes the value of 0 if an individual gave birth within 10 months of marriage or 1 if an individual gave birth after 10 months of marriage).

### 2.3. The Risk Factors/Predictors of Birth Length Status

The status of the outcome variable, introduced in the previous section, depends on certain risk factors. These risk factors predict the status of the outcome variable. In this section, we introduce the risk factors for the birth length status. These risk factors will be used in the data analyses section to account for their influence on the status of the outcome variable.

The study adjusted for the effect of partner's educational attainment on birth length status (which takes a value of 0 if no education, 1 if primary education, 2 if secondary, and 3 if higher education). We also adjusted for the effect of pregnancy termination birth length status, where respondent was asked if she ever terminated pregnancy (which takes a value of 0 if no and 1 if yes). Other risk factors included in the birth length status model are geographical location (takes a value of 0 if an individual live in a rural area or 1 if an individual live in urban area), oral contraceptive usage (takes a value of 0 if no or 1 if yes), anaemia status (takes a value of 0 if no anaemia or 1 if yes), husband stays at home (takes a value of 0 if no or 1 if yes), and beating justified if refuses to have sex, if burns food, argues with husband, neglects child, or goes out without informing husband (takes a value of 0 if no or 1 if yes). We also adjust for the effect of some continuous risk factors such as age at marriage, haemoglobin level, and weight.

Table 1 presents descriptive statistics of the variables used (outcome and predictor variables) in this study. It can be observed that high proportion (approximately 68%) of the respondents give birth to their first child beyond 10 months after marriage, with approximately 68% of the respondents living in rural areas. Only 20% of the respondents have ever terminated pregnancy, and minority (approximately 27%) of these respondents use oral contraceptives. The descriptive statistics also suggest that approximately 47% of the respondents have anaemia and majority (78%) of the respondents' husbands do not live in the same home with them. We observed that majority (50%) of the respondents have secondary education followed by no education (with 35%), and only 6% have higher education. Respondents were also asked whether beating of wife is justified in situations such as going out without husband's notice, neglect of a child, arguing with the husband, refusal to have sex with the husband, and burning of food. From the descriptive statistics, majority (65%, 61%, 65%, 75%, and 83%, respectively) of the respondents (in each situation) reported that beating is not justified. However, approximately 35%, 39%, 34%, 25%, and 16% of the respondents reported that beating is justified if wife goes out without husband's notice, neglects child, argues with the husband, refuses to have sex with the husband, and burns of food, respectively. The mean weight, haemoglobin level, age at marriage, and age at first sex are presented in Table 1, where the mean age of the respondents is approximately 20 with average age at first sex as 17. This means that respondents begin sex at least two years prior to marriage.

### 2.4. Statistical Analysis

In this section, we discuss some selected statistical approaches/tools that will allow us to investigate the relationship between the outcome variable (birth length status) and the predictors of birth length status.

In this study, we used two statistical approaches to analyse the data on birth records. First, we used the Chi-Square test statistic\citep{[18] chi} to investigate the association between the outcome variable of interest and the predictors of birth length status, introduced in the previous section. The purpose for this analysis is to search for the existence of variables that are potential predictors of the status of the outcome variable. This means that variables that are found to have or likely to have significant association with the birth length status will be considered for further analyses.

In the further analyses, we used the logistic regression model [17, 19–23] to establish the relationship and to estimate the effect of the predictor variables on birth length status. The general form of a logistic regression model can be written as

(1) $$\begin{array}{c} \text{logit}\left[\mathrm{Pr}\left(y_{i}\;|\;X,\beta \right)\right]=\text{logit}\left(p\right)=\log\left(\frac{p}{1-p}\right)=\beta_{0}+\beta_{1}X_{1}+\cdots +\beta_{p}X_{p}, \end{array}$$

where *X*_1_, ⋯, *X*_*p*_ are the risk factors/predictors; *β*_0_, ⋯, *β*_*p*_ are parameter estimates representing the effects of their corresponding risk factors on the dichotomous response variable (birth length status); *y*_*i*_ is the outcome which equals to 1 if respondent *i* gives birth to her first child after 10 months of marriage and 0 if within 10 months; **X** is a design matrix for the predictors; and **β** is a vector of the parameter estimates. Also, *p* is the probability that a respondent gives birth after 10 months of marriage and *p*/(1 − p) is the odds of the outcome variable among those who exposed to the predictors relative to those who are not exposed to the same predictors. So in effect, the **β**  is the log odds ratio of the birth length status for those who are exposed to the predictors relative to those who are not exposed.

We use this model (1) to predict the response probability for an individual for which the values of the predictors in the model are observed. In order to determine the predicted probability, we need to back-transform using

(2) $$\begin{array}{c} \hat{p}=\frac{\left({\hat{\beta }}_{0}+{\hat{\beta }}_{1}X_{1}+\cdots +{\hat{\beta }}_{p}X_{p}\right)}{\left(1+{\hat{\beta }}_{0}+{\hat{\beta }}_{1}X_{1}+\cdots +{\hat{\beta }}_{p}X_{p}\right)}, \end{array}$$

where ${\hat{\dddot{\beta }}}_{1},\cdots ,{\hat{\dddot{\beta }}}_{p}$ are estimators of *β*_1_, ⋯, *β*_*p*_, respectively. These predicted probabilities are used to classify individuals into either those who actually give birth to their first child within 10 months after marriage or after 10 months of marriage. This approach is known as classification \citep{van2004methodology,collett2002modelling}. Thus, to assign an individual to one of the two groups on the basis of the predicted response probabilities, we need to identify a “threshold” value, *π*_0_ , in such a way that if $\hat{p}\geq \pi_{0}$, then an individual should be classified into group 1 and if $\hat{p}<\pi_{0},$then an individual should be classified into group 2. This means that *π*_0_ = 0.5$ if the two groups are symmetrical. Also, *π*_0_ can be determined from the observed data, where *π*_0_ is chosen so as to either minimize the overall proportion of misclassification or to compromise between the minimization of the 2 misclassification probabilities (that is, the probability of allocating an individual to group 1 when he/she should be in group 2 and vice versa).

When our two groups refer to those who give birth to their first child within 10 months and those who give birth to their first child after 10 months of marriage, we can summarize the relationship between the true situation and the prediction as shown in Table 2. Using the information in Table 2, we defined sensitivity as the percentage of individuals who give birth after 10 months of marriage are classified as actually giving birth to their first child after 10 months of marriage. This implies that sensitivity is given by a/(a + c) × 100. We also define specificity as the percentage of individuals who give birth to their first child within 10 months of marriage over those classified as those who do not actually give birth after 10 months of marriage (give birth within 10 months of marriage). Thus, specificity is given by *b*/(*b* + *d*) × 100 [20]. We define probabilities of the two misclassification situations as (1) *b*/(*b* + *d*) = 1 − SPEC (expressed as proportion), which represents the probability of positive prediction given that an individual gives birth to the first child within 10 months of marriage and (2) *a*/(*a* + *c*) = 1‐$SEN (expressed as proportion), which represents the probability of negative prediction given that an individual gives birth to the first child after 10 months of marriage. These methods are often used in disease status prediction and classification, and depending on the nature of the disease, one of these misclassifications may be more serious than the other. More often, the focus is to minimize the probability of false negative, which is equivalent to maximizing sensitivity.

In this study, we use the Receiver Operating Characteristic (ROC) curve [24, 25] to determine the predictive power of the model fitted to the birth records data. The ROC curve is a plot of sensitivity versus 1-specificity as the cutoff *π*_0_varies. Since we classify an individual as one who gives birth to their first child after 10 months of marriage if $\hat{p}\geq \pi_{0}$ and one within 10 months if $\hat{p}<\pi_{0}$, the number of positive predictions will increase as the threshold decreases. This gives an indication that sensitivity will increase with decreasing *π*_0_ and 1-specificity will increase with decreasing *π*_0_. On the other hand, when sensitivity is equal to 1-specificity, the probability of positive prediction given that an individual gives birth to their first child after 10 months is comparable to the probability of positive prediction given that an individual gives birth to a child within 10 months. In this situation, the model has no predictive power. For a highly predictive model, we want sensitivity to be much bigger than 1-specificity (we want sensitivity to increase much faster than 1-specificity) as *π*_0_ goes from 1 to 0. Statistical analyses in this study were carried out using the STATA version 14.1 software ([26, 27].

## 3. Results

In this section, we present and discuss the results obtained using the Chi-Square test statistic. Here, our focus is the magnitude and significance of statistical association between the outcome variable (birth length status) and the various predictors. We also present and provide interpretation of the results from the logistic regression model (1).

### 3.1. Results from the Chi-Square Test Statistic

In Table 3, we present the results of the Chi-Square test of association between the outcome variable (birth length status) and the predictors. The purpose of this exercise is to identify variables that are more likely to predict the status of the outcome variable. The Chi-Square test statistic results presented show that (1) geographical location (*Χ*_(1, 5%)_^2^ = 4.61, *p* value = 0.032), (2) partner's educational level (*Χ*_(3, 5%)_^2^ = 10.19, *p* value = 0.017), (3) beating justified if wife neglects child (*Χ*_(1, 5%)_^2^ = 9.63, *p* value = 0.002), (4) beating justified if wife argues with the husband (*Χ*_(1, 5%)_^2^ = 13.94, *p* value = 0.001), (5) beating justified if wife burns food (*Χ*_(1, 5%)_^2^ = 8.37, *p* value = 0.004), (6) age of the respondent (*t* = ‐13.13, *p* value = 0.001), and (7) ever terminated pregnancy (*Χ*_(1, 5%)_^2^ = 5.08, *p* value = 0.024) are significantly associated with the outcome variable and hence are potential predictors of the status birth length. However, predictors such as (1) contraceptive use (*Χ*_(1, 5%)_^2^ = 2.10, *p* value = 0.148), (2) anaemia status (*Χ*_(1, 5%)_^2^ = 1.84, *p* value = 0.18), (3) husband at home status (*Χ*_(1, 5%)_^2^ = 2.10, *p* value = 0.15), (4) beating justified if wife goes out without husband's notice (*Χ*_(1, 5%)_^2^ = 3.24, *p* value = 0.072), (5) beating justified if wife refuses to have sex (*Χ*_(1, 5%)_^2^ = 3.11, *p* value = 0.078), (6) weight (*t* = −1.05, *p* value = 0.293), (7) age at first sex (*t* = 1.13, *p* value = 0.259), and (8) haemoglobin level (*t* = ‐0.04, *p* value = 0.971) of the respondent are not statistically significant, and hence, they are not reported in the results of our further analyses (using the logistic regression model).

### 3.2. Results from the Logistic Regression Model

In this section, we build a logistic regression model to estimate effects and to assess the significance of the predictors on the status of the outcome (birth length). We select the best predictors/model by going through the following four steps. However, this can also be done using an automatic stepwise variable selection procedure given in the STATA code attached.

First, we fitted a logistic model (Model A) with all the predictors in Table 3. Highly statistically insignificant predictors such as whether husband stays at home or not (odds ratio (OR) = 0.031, *p* value = 0.651), beating justified if wife refuses to have sex with husband (OR = 0.034, *p* value = 0.693), and weight (*p* value = 0.747 with approximately zero estimate) are removed in the subsequent analyses. In the second analyses, we fitted a logistic regression model (Model B) without the highly statistically insignificant predictors in Model A. In this model, the predictor, beating justified if wife burns food was highly insignificant (OR = −0.074, *p* value = 0.389) and was removed in the subsequent analysis. So, in the third model (Model C), we fitted a logistic regression model to the birth length status data without the highly insignificant predictors in Models A and B. However, predictors such as geographical location (OR = 0.101, *p* value = 0.112), primary (OR = 0.112*p* value = 0.270) and secondary education (OR = 0.038, *p* value = 0.252), and haemoglobin level (OR = 0.005, *p* value = 0.070) of the respondents were still insignificant in analyses under Model C and hence were removed in the subsequent analyses. In the fourth model, Model D, all the insignificant predictors under Models A, B, and C were removed.

We stored/saved estimate results from Models A, B, C, and D and then compared the performance of these models using their respective AICs shown in Table 4. The model with the lowest AIC or BIC and the smallest number of parameters is the best fitting model for predicting/estimating the status of birth length. So it can be observed in Table 4 that the best fitting model is Model D since it has the lowest AIC and BIC as well as small number (8) of parameters. It is important to note that Model D and Model C have approximately equal AIC values, but Model D is selected as the best model because it has the lowest number of parameters (parsimonious model) and lowest BIC as well.

The results of the best fitting Model D are presented in Table 5. Here, the unadjusted OR and adjusted OR are presented. The results show that respondents who have ever terminated pregnancy are more likely (OR = 0.178, 95%CI = 0.044, 0.312) to deliver their first child after 10 months of marriage relative to those who have never terminated pregnancy. Wives whose husbands have higher education are less likely (OR = −0.162, 95%CI = −0.236, -0.088) to give birth to their first child after 10 months of marriage (that is, they are more likely to give birth to their first child within 10 months of marriage) compared with those who have no education.

We observed that women who reported that beating is justified if she goes out without her husband's notice are more likely (OR = 0.466, 95%CI = 0.305, 0.628) to give birth to their first child after 10 months of marriage. This finding appears to suggest that wives are more likely to violate this order, and hence, there may be frequent misunderstanding between partners, which may subsequently lead to infrequent sexual intercourse which may delay conception among such women. On the contrary, wives who reported that beating is justified if she neglects child are more likely (OR = −0.305, 95%CI = −0.461, 0.149) to give birth to their first child within 10 months of marriage. This finding which may suggest that wives who believe that husbands care much about their children are more willing to give first within the shortest possible time. Wives who reported that beating is justified when she argues with her husband are less likely (OR = −0.301, 95% = −0.451, -0.152) to give birth to their first child after 10 months of marriage (that is, they are more likely to give birth within 10 months of marriage) relative to wives who reported that beating is not justified if she argues with her husband. This suggests that such wives are more likely to offer sex to their husband without arguing/refusing anytime they demand and hence more likely to conceive within 1 month after marriage. These findings, overall, appear to give an indication that various forms of social violence are key factors in determining how long a woman stays in a marriage before giving birth to the first child and, possibly, the next child and so on.

We found that for every unit increase in the age of the respondent at marriage, there is a 0.125 increase in the likelihood of giving birth to the first child after 10 months of marriage. This finding is probably due to a higher proportion (28%) of contraceptive use among older-age (>18 years) wives relative to approximately 26% of contraceptive use among the younger-age (≤18) wives. The results show that for every unit increase in the age of respondent at first sex, there is a 0.029 decrease in the likelihood of giving birth to the first child after 10 months in marriage. This suggests that if a woman stays longer to have her first sex, then such woman is more likely to have more sex in marriage and more likely to conceive within 1 month of marriage. Though not general, but it is more likely that younger wives and those who stay longer to have their first sex may be more willing or have much desire to have sex, hence more likely to conceive within 1 month of their marriage.

### 3.3. Assessing the Predictive Power of Model D

**Figure 1:**
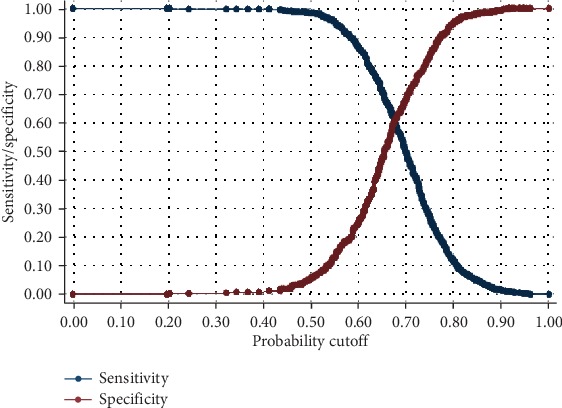
Graph of sensitivity and specificity versus different cutoff values *π*_0_ from 0 to 1.

In this section, we assess the predictive power of Model D in predicting probabilities for birth length status using the Receiver Operating Characteristic (ROC) curve discussed in Section 2. To assess the power of Model D in classification of individuals according to the proportion of individuals who give birth to their first child after 10 months of marriage given that they actually give birth after 10 months of marriage, we first determine the cutoff *π*_0_ value using Figure 1. This graph of sensitivity and specificity versus the different values of the threshold *π*_0_can assist in deciding on an optimal value of *π*_0_. For instance, when *π*_0_ = 0.65 gives higher (70.94%) sensitivity and lower (47.39%) specificity. When *π*_0_ = 0.60, sensitivity is higher at 85.16% and specificity is lower at 25.80%. This means that decreasing *π*_0_ value leads to increasing sensitivity. Since we want sensitivity to increase much faster, we may use a cutoff value *π*_0_ = 0.60$ or *π*_0_ = 0.55$ which gives sensitivity as 95.11% and specificity as 12.78%. For instance, using *π*_0_ = 0.63$ produces the following classifications presented in Tables 6 and 7. These results give an indication sensitivity (the probability that an individual gives birth to the first child after 10 months of marriage given that such individual actually gives birth after 10 months) is 77.78%. Also, specificity (the probability that an individual gives birth to the first child within 10 months of marriage given that such individual actually gives birth within 10 months) is 36.69%. Positive and negative predictive values are 72.61% and 43.34%, respectively. The probability of misclassifications for positive and negative rates are 27.39% and 56.66%, respectively.

**Figure 2:**
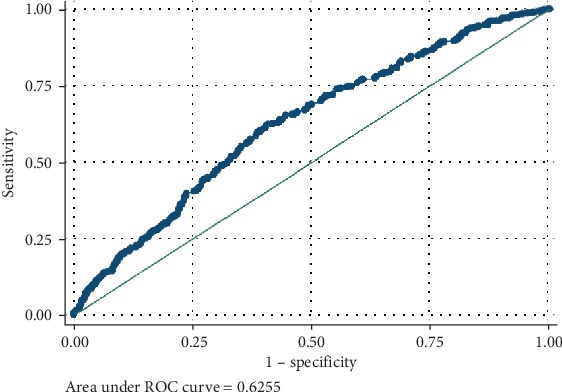
Graph of sensitivity versus 1-specificity.

We now assess the predictive power of Model D using the ROC curve to estimate the area under the curve. The ROC curve is a plot of sensitivity versus 1-specificity. This means that when sensitivity = 1 − specificity, the area under the curve is 50%, corresponding to no predictive power. If sensitivity increases faster than 1-specificity, the more bowed is the ROC curve and the bigger is the predictive power of the model. This corresponds to a larger area under the ROC curve. Hence, the shape of the ROC curve and the area under the curve is indications of the predictive power of the model. Hence, the ROC curve displayed in Figure 2 gives an indication that the area under the curve is approximately 63%, indicating that the model has bigger predictive power.

## 4. Conclusion

In this paper, we investigated the effects of various risk factors on the first child's birth length after marriage. Birth length in this study is a dichotomous variable coded as 1 if an individual gives birth to her first child after 10 months of marriage, and 0 if an individual gives birth to her first child within 10 months of marriage. The study used birth record data from the 2014 Ghana Demographic and Health Survey (2014 GDHS). Some of the variables have missing values (Committee for Medicinal Products for Human Use (CHMP) & [16, 28], 2019a; [17, 29]) and hence excluded from the analyses. This means that the analyses in this paper are restricted to 6,525 complete cases [16, 17] of individuals with no missing values with 18 risk factors of birth length status. STATA code for statistical analyses in this paper is attached in the Appendix.

We assessed the effects of the various risk factors on the status of the dichotomous birth length variable using the logistic regression model [19, 21, 23]. The purpose of this exercise is to identify the best risk factors (predictors) of birth length status. To do this, we first used the Chi-Square test statistic [22] to investigate the association between the predictors and the outcome birth length status. The Chi-Square results show that geographical location, ever terminated pregnancy, partner's educational level, beating of wife justified if wife goes out without husband's notice, neglects child, argues with her husband, burns food, and age of respondent are significantly associated with the outcome birth length. This implies these predictors are more likely to predict the status of the outcome. The Chi-Square test statistic results represent a form of univariate analysis assessing the effect of individual predictors on the outcome. So in our subsequent analyses, we used the logistic regression model to allow us to include many/all the predictors to assess their effects on the status of the birth length.

We build our regression model for the dichotomous birth length variable by fitting four logistic regression Models A, B, C, and D (explained in Section 3). Results from these models were compared, using their respective Akaike Information Criterion (AIC), and the best fitting model for the data selected. The AIC is an estimator which measures the relative quality of statistical models for a given set of data. Model D was selected as the besting fitting model since it has the lowest AIC and Bayesian Information Criterion (BIC) as well as parsimonious (smallest number of parameters/variables). We then assessed the predictive power of Model D in predicting the probabilities of the status of birth length. We achieved this using the Receiver Operating Characteristic (ROC) curve. The ROC curve is a plot of sensitivity versus 1-specificity. With an optimal cutoff value of 0.63, Model D produces a higher sensitivity relative specificity, and specificity (the probability that an individual gives birth to the first child within 10 months of marriage given that such individual actually gives birth within 10 months) is 36.69%. The probability of misclassifications for positive and negative rates is 27.39% and 56.66%, respectively. So with the ROC curve, when sensitivity = 1 − specificity, the model has no predictive power (and area under the curve is 50%). The ROC curve in this study gives an indication that the area under the curve is approximately 64%, which indicates that Model D has higher predictive power.

The results from Model D showed that respondents who have ever terminated pregnancy are more likely to give birth to their first child after 10 months of marriage and wives whose husbands have higher education are less likely to give birth to their first child after 10 months of marriage. It was observed that women who reported that beating is justified if she goes out without her husband's notice are more likely to give birth to their first child after 10 months of marriage. This suggests that wives are more likely to violate this order, and hence, there may have frequent misunderstanding between partners. This will subsequently lead to infrequent sexual intercourse which may delay conception among such women. On the other hand, we found that among wives who reported that beating is justified if she neglects child are more likely to give birth to their first child within 10 months of marriage. This finding suggests that wives who believe that husbands care much about their children are more willing to give first within the shortest possible time. Wives who reported that beating is justified when she argues with her husband are less likely to give birth to their first child after 10 months of marriage. The finding suggests that such wives are more likely to offer sex to their husband without arguing/refusing anytime they demand and hence more likely to conceive within 1 month after marriage. These findings, overall, appear to give an indication that various forms of social violence are key factors in determining how long a woman stays in a marriage before giving birth to the first child and, possibly, the next child and so on.

The results in this study also showed that there is an increase in the likelihood of giving birth to the first child after 10 months of marriage for every unit increase in the age of respondent at marriage. This could be explained by the higher proportion of contraceptive use among older-age (>18 years) wives relative to a lower contraceptive use among the younger-age (≤18) wives. Also, for every unit increase in the age of respondent at first sex, there is a decrease in the likelihood of giving birth to the first child after 10 months in marriage, which appears to suggest that if a woman stays longer to have her first sex, then such woman is more likely to have more sex in marriage and more likely to conceive within 1 month of marriage. Though not general, but it is more likely that younger wives and those who stay longer to have their first sex may be more willing or may have much desire to have sex, hence more likely to conceive with 1 month of the marriage.

Our study results suggest that for conception within 1 month of marriage or to give birth to the first child within 10 months of marriage, wives and husbands should have frequent sex. The results also appear to suggest that any negative social behaviour or policies, on the part of the husband or society, that will reduce the likelihood of having frequent sex with the wife must be discouraged. Women who wish to have children are encouraged to desist from termination of pregnancy at any time of their life since this act has the likelihood of reducing chances of conception for such women. Based on our results, we also encourage husbands to openly express their desire and love for their children since this increases the likelihood of wives' desire to give birth. This leads to frequent sex, which then reduces conception time, and hence, child birth within the shortest possible time.

## Figures and Tables

**Table 1:** Descriptive statistics of the outcome variable (birth length status) and the predictor variables.

Variable	*N*	% or mean
Birth length status		
1–10 months	2,066	31.66
*>*10 months	4,459	68.34
Geographical location		
Rural	4,429	67.88
Urban	2,096	32.12
Ever terminated pregnancy?		
No	5,182	79.42
Yes	1,343	20.58
Oral contraceptive use		
No	4,732	72.52
Yes	1,793	27.48
Anaemia status		
No	3,479	53.32
Yes	3,046	46.68
Husband stays at home		
No	5,099	78.15
Yes	1,426	21.85
Partner's educational level		
No education	2,264	34.70
Primary	608	9.32
Secondary	3,266	50.05
Higher	387	5.93
Beating justified if goes out without husband's notice?		
No	4,212	64.55
Yes	2,313	35.45
Beating justified if neglects child?		
No	3,951	60.55
Yes	2,574	39.45
Beating justified if argues with husband?		
No	4,266	65.38
Yes	2,259	34.62
Beating justified if refuses to have sex with husband?		
No	4,916	75.34
Yes	1,609	24.66
Beating justified if burns food?		
No	5,434	83.28
Yes	1,091	16.72
Mean age of respondents	6,525	19.65
Mean age at first sex	6,525	17.38
Mean weight of respondents	6,525	591.841
Mean weight of haemoglobin level	6,525	119.51

**Table 2:** Prediction and classification of birth length status.

Prediction	Birth after 10 months	Birth within 10 months	Total predictions
Positive prediction	True +Ve (a)	False +Ve (b)	Number of +Ve predictions (a + b)
Negative prediction	False -Ve (c)	True -Ve (d)	Number of -Ve predictions (c + d)
True totals	After 10 months (a + c)	Within 10 months (b + d)	

**Table 3:** A Chi-Square test of the association between outcome variable and the predictors.

Variable	Birth length status	
	Within 10 months	After 10 months
Geographical location	*χ* ^2^ = 4.61, *p* value = 0.032	
Rural	1,440	2,989
Urban	626	1,470
Ever terminated pregnancy?	*χ* ^2^ = 5.08, *p* value = 0.024	
No	1,675	3,507
Yes	391	952
Use contraceptives?	*χ* ^2^ = 2.10, *p* value = 0.148	
No	1,474	3,258
Yes	592	1,201
Has anaemia?	*χ* ^2^ = 1.84, *p* value = 0.18	
No	1,127	2,352
Yes	939	2,107
Husband lives at home?	*χ* ^2^ = 2.10, *p* value = 0.15	
No	1,637	3,462
Yes	429	997
Partner's educational level	*χ* ^2^ = 10.19, *p* value = 0.017	
No education	729	1,535
Primary	198	410
Secondary	992	2,274
Higher	147	240
Beating justified if wife goes out without husband's notice?	*χ* ^2^ = 3.24, *p* value = 0.072	
No	1,366	2,846
Yes	700	1,613
Beating justified if wife neglects child?	*χ* ^2^ = 9.63, *p* value = 0.002	
No	1,194	2,757
Yes	872	1,702
Beating justified if wife argues with husband?	*χ* ^2^ = 13.94, *p* value = 0.001	
No	1,284	2,982
Yes	782	1,477
Beating justified if wife refuses sex?	*χ* ^2^ = 3.11, *p* value = 0.078	
No	1,528	3,388
Yes	538	1,071
Beating justified if wife burns food?	*χ* ^2^ = 8.37, *p* value = 0.004	
No	1,680	3,754
Yes	386	705
Age of respondent at first sex	*t*-value = 1.13, *p* value = 0.259	
	2066	4459
Weight of respondent	*t*-value =‐1.05, *p* value = 0.293	
	2066	4459
Age of respondent	*t*-value = -13.13, *p* value = 0.001	
	2066	4459
Haemoglobin level of respondent	*t*-value = -0.04, *p* value = 0.971	
	2066	4459

**Table 4:** Comparison of Models A, B, C, and D.

Model	Number of observations	Degrees of freedom	AIC	BIC
A	6525	18	7892.752	8014.853
B	6525	15	7887.199	7988.950
C	6525	14	7885.940	7980.908
D	6525	8	7886.447	7940.714

**Table 5:** Unadjusted and adjusted odds ratio and 95% confidence interval (95% CI): logistic regression model.

Covariates	Unadjusted OR		Adjusted OR	
	Odds ratio	95% CI	Odds ratio	95% CI
Ever terminated pregnancy?				
No	1 (reference)		1 (reference)	
Yes	0.151	(0.020, 0.282)	0.178	(0.044, 0.312)
Partner's educational level				
No education	1 (reference)		1 (reference)	
Higher education	-0.099	(-0.170, -0.028)	-0.162	(-0.236, -0.088)
Beating justified if wife goes out without husband's notice?				
No	1 (reference)		1 (reference)	
Yes	0.101	(-0.009, 0.210)	0.466	(0.305, 0.628)
Beating justified if wife neglects child?				
No	1 (reference)		1 (reference)	
Yes	-0.168	(-0.274, -0.062)	-0.305	(-0.461, -0.149)
Beating justified if wife argues with husband?				
No	1 (reference)		1 (reference)	
Yes	-0.207	(-0.315, -0.098)	-0.301	(-0.451, -0.152)
Age of respondent at marriage	0.109	(0.092, 0.125)	0.125	(0.107, 0.143)
Age at first sex	-0.006	(-0.016, 0.004)	-0.029	(-0.041, -0.018)

**Table 6:** Prediction and classification of birth length status.

Prediction	Birth after 10 months (B)	Birth within 10 months (∼B)	Totals
+Ve	3468	1308	4776
-Ve	991	758	1749
Total	4459	2066	6525

**Table 7:** Prediction and classification of birth length status.

Sensitivity	Pr(+|B)	77.78%
Specificity	Pr(−|∼B)	36.69%
Positive predictive value	Pr(B|+)	72.61%
False +rate for true ∼B	Pr(+|∼B)	63.31%
False -rate for true B	Pr(−|B)	22.22%
False +rate for classified	Pr(∼B|+)	27.39%
False -rate for classified-	Pr(B|−)	56.66%

## Data Availability

We used data from the 2014 Ghana Demographic Health Survey.

## Appendix

∗STATA annotated code for analyses of birth length status data∗

∗Descriptive statistics for dichotomous outcome and categorical predictors∗

tab birthlength

tab residence

tab abortionever

tab ocuse

tab analevel

tab husbandhome

tab partnaedulevel

tab beatifgoout

tab beatifnegchld

tab beatifargue

tab beatifrefussex

tab beatifburnfood

∗Descriptive statistics of continous predictors

mean agefirstsex

mean haemlevel

mean weight

mean age

∗Chi-Square test assocciation between dichotomous outcome categorical predictors∗

tabulate residence birthlength, chi2

tabulate abortionever birthlength, chi2

tabulate ocuse birthlength, chi2

tabulate analevel birthlength, chi2

tabulate husbandhome birthlength, chi2

tabulate partnaedulevel birthlength, chi2

tabulate beatifgoout birthlength, chi2

tabulate beatifnegchld birthlength, chi2

tabulate beatifargue birthlength, chi2

tabulate beatifrefussex birthlength, chi2

tabulate beatifburnfood birthlength, chi2

∗Compare means continous predictors for different groups∗

ttest agefirstsex, by(birthlength)

ttest haemlevel, by(birthlength)

ttest weight, by(birthlength)

ttest age, by(birthlength)

∗Now build a logistic model∗

∗Since partner's educational level has more than two levels, we creat dummy variables as:∗

gen Primeducation=0

replace Primeducation=1 if (partnaedulevel==1)

gen Secondeducation=0

replace Secondeducation=2 if (partnaedulevel==2)

gen Highereducation=0

replace Highereducation=3 if (partnaedulevel==3)

∗Birth length model with all predictors:∗

logit birthlength residence abortionever ocuse analevel husbandhome Primeducation Secondeducation Highereducation beatifgoout beatifnegchld beatifargue beatifrefussex beatifburnfood age agefirstsex weight haemlevel

∗Store estimates/results in A∗

est store A

∗Birth length status model with husbandhome,beatifrefussex, and weight variables removed∗

logit birthlength residence abortionever ocuse analevel Primeducation Secondeducation Highereducation beatifgoout beatifnegchld beatifargue beatifburnfood age agefirstsex haemlevel

∗Store estimates/results in B∗

est store B

∗Now compare model estimates in A with B∗

lrtest A B,stats

∗Birth length status model with husbandhome,beatifrefussex, weight, and beatifburnfood variables removed∗

logit birthlength residence abortionever ocuse analevel Primeducation Secondeducation Highereducation beatifgoout beatifnegchld beatifargue age agefirstsex haemlevel

∗Store estimates/results in C∗

est store C

∗Birth length status model with husbandhome,beatifrefussex, weight, Primeducation, Secondeducation, analevel, haemlevel and beatifburnfood variables removed∗

logit birthlength abortionever Highereducation beatifgoout beatifnegchld beatifargue age agefirstsex

∗Store estimates/results∗

est store D

∗Now compare model estimates ∗

lrtest A B,stats

lrtest A C,stats

lrtest A D,stats

∗Now compare model estimates∗

lrtest B C,stats

lrtest B D,stats

∗Now compare model estimates ∗

lrtest C D,stats

∗Stepwise logistic regression model∗

sw, pe(.05) lr:logit birthlength residence abortionever ocuse analevel husbandhome Primeducation Secondeducation Highereducation beatifgoout beatifnegchld beatifargue beatifrefussex beatifburnfood age agefirstsex weight haemlevel

∗Unadjusted odds ratio∗

logit birthlength abortionever

logit birthlength Highereducation

logit birthlength beatifgoout

logit birthlength beatifnegchld

logit birthlength beatifargue

logit birthlength age

logit birthlength agefirstsex

∗Classification: Determin cutoff value in range 0-1∗

logit birthlength abortionever Highereducation beatifgoout beatifnegchld beatifargue age agefirstsex

lsens,xlabel(0 0.1 0.2 0.3 0.4 0.5 0.6 0.7 0.8 0.9 1.0) ylabel(0 0.1 0.2 0.3 0.4 0.5 0.6 0.7 0.8 0.9 1.0)

∗ROC curve for 0.65

logit birthlength abortionever Highereducation beatifgoout beatifnegchld beatifargue age agefirstsex

lstat,cutoff(0.65)

predict p,p

roctab birthlength p, graph

∗ROC curve for 0.60

logit birthlength abortionever Highereducation beatifgoout beatifnegchld beatifargue age agefirstsex

lstat,cutoff(0.63)

drop p

predict p,p

roctab birthlength p, graph

∗ROC curve for 0.55

logit birthlength abortionever Highereducation beatifgoout beatifnegchld beatifargue age agefirstsex

lstat,cutoff(0.55)

drop p

predict p,p

roctab birthlength p, graph
